# Mixed Micelles of Sodium Cholate and Sodium Dodecylsulphate 1:1 Binary Mixture at Different Temperatures – Experimental and Theoretical Investigations

**DOI:** 10.1371/journal.pone.0102114

**Published:** 2014-07-08

**Authors:** Balázs Jójárt, Mihalj Poša, Béla Fiser, Milán Szőri, Zita Farkaš, Béla Viskolcz

**Affiliations:** 1 Department of Chemical Informatics, Faculty of Education, University of Szeged, Szeged, Hungary; 2 Department of Pharmacy, Faculty of Medicine, University of Novi Sad, Novi Sad, Serbia; Jacobs University Bremen, Germany

## Abstract

Micellisation process for sodium dodecyl sulphate and sodium cholate in 1∶1 molar ratio was investigated in a combined approach, including several experimental methods and coarse grained molecular dynamics simulation. The critical micelle concentration (cmc) of mixed micelle was determined by spectrofluorimetric and surface tension measurements in the temperature range of 0–50°C and the values obtained agreed with each other within the statistical error of the measurements. In range of 0–25°C the cmc values obtained are temperature independent while cmc values were increased at higher temperature, which can be explained by the intensive motion of the monomers due to increased temperature. The evidence of existing synergistic effect among different constituent units of the micelle is indicated clearly by the interaction parameter (β_1,2_) calculated from cmc values according to Rubingh. As the results of the conductivity measurements showed the negative surface charges of the SDS-NaCA micelle are not neutralized by counterions. Applying a 10 µs long coarse-grained molecular dynamics simulation for system including 30-30 SDS and CA (with appropriate number of Na^+^ cations and water molecules) we obtained semi-quantitative agreement with the experimental results. Spontaneous aggregation of the surfactant molecules was obtained and the key steps of the micelle formation are identified: First a stable SDS core was formed and thereafter due to the entering CA molecules the size of the micelle increased and the SDS content decreased. In addition the size distribution and composition as well as the shape and structure of micelles are also discussed.

## Introduction

Bile acids are steroids synthesized in the liver of invertebrates. They are mostly hydroxyl (OH) derivatives of 5β-cholanoic acid and are in anionic form under physiological conditions. These bile acid anions are biplanar surface active materials: the α (concave) side of the steroid skeleton is hydrophilic, and totally separated from the β convex hydrophobic side [1]–[4]. Among the anionic bile acids, 3α,7α,12α-trihydroxy-5β-cholanoic acid (CA) is the most representative structure of these biplanar surfactants [1]. The *cmc* value (6.5 mM to 16 mM) of its sodium salt, NaCA, depends on the determination method used as well as experimental condition (e.g. pH, and ionic strength) [1], [3]. Similarly, the aggregation number (average number of molecules in a micelle) of pure NaCA (3 to 16) is also highly influenced by the environmental parameters [2], [3]. The aggregation number of pure NaCA micelle is 2–4 at the *cmc* value and the driving force of the aggregation is hydrophobic interactions (Small-Kawamura model) [5], [6]. At higher concentrations not only hydrophobic, but hydrophilic interactions can also play a possible role between cholate molecules in micelles [6]. The regiochemistry of the steroid skeleton with the OH groups provides a good hydrophilic binding position for different pharmacologically active molecules through hydrogen bonds. These accessible OH groups also provide other binding sites at open hydrophilic pockets of the micelle. These opposing effects can result in an altered bioavailability of the drug (depot effect, promotor in the pass through the blood-brain barrier, solubilization effect) [7]–[11]. Unfortunately, the relatively small size of the bile acid micelles restricts their usage as an appropriate drug carrier. One of the possible ways to increase the aggregation number is to form mixed micelles. SDS is a classical surfactant having a long hydrophobic tail and a polar head with a *cmc* value of 8.5 mM. The shape of the SDS micelles is spherical-elliptical and the aggregation number can be as high as 120. SDS is widely used in the pharmaceutical industry as a solubilizer and emulsifier [12].

Previously, the aggregation of pure SDS and pure NaCA were studied by various models, including united atom [13], all atom [14], [15] and coarse-grained models [16], but the properties of their mixtures, and the mechanism of their aggregation are not yet understood. Therefore, we investigated the micelle formation in the binary mixture of NaCA and SDS in 1∶1 mole fraction. The *cmc* value of the binary mixture is determined in the temperature range from 0 to 50°C by spectrofluorimetry and surface tension measurements. For the molecular level of understanding of the observed phenomena, coarse-grained molecular dynamics simulation was also performed at 27°C for the binary mixture.

## Materials and Methods

All chemicals used in the experiments were obtained from Sigma, the purity of NaCA and SDS was 99.98% and the molar ratio was 1∶1 (molar fraction (α)  = 0.5).

### Spectrofluorimetric measurements

Fluorescence measurements were carried out using an Agilent Cary Eclipse Fluorescence Spectrophotometer using pyrene as a fluorescence probe molecule. All solutions of the binary mixture of surfactants were prepared using pyrene saturated water. Fluorescence emission spectra of these solutions were recorded employing an excitation wavelength of 334 nm. The intensities of first (I_1_) and third (I_3_) vibronic bands of the pyrene emission spectrum were measured at 373 and 384 nm, respectively. The I_1_/I_3_ ratio was monitored as a function of total surfactant concentration (*c*) resulting fluorimetric titration curve at every 5°C between 0 and 50°C. The *cmc* was determined by fitting Boltzmann function to the fluorimetric titration curve. Trials were repeated (*n*) seven times for reproducibility. The relative error of the *cmc* determination never exceeded 3%.

### Surface tension measurements

Surface tension measurements were carried out on aqueous solutions of sodium salts of binary mixtures surfactants. Surface tension was measured by a Krüss tensiometer (Germany) using a du Nouy ring method. All measurements were repeated seven times (*n* = 7) at each temperature. The relative error of the *cmc* determined by surface extension method did not exceed 3%.

### Conductivity measurements

The goal of the conductometry measurement was to determine the fraction of counterion binding to the micelle. Conductivity was measured by gradual dilution of surfactant solutions with deionised water in the cases of aqueous solutions of pure sodium salts of SDS and the binary mixtures. The data were acquired using a Consort C 860 conductometer. The cell containing solutions was immersed in a water bath, controlling the temperature variation at ±0.1°C. The temperature was kept constant at 25°C.

### Molecular dynamics simulations

**Figure 1:**
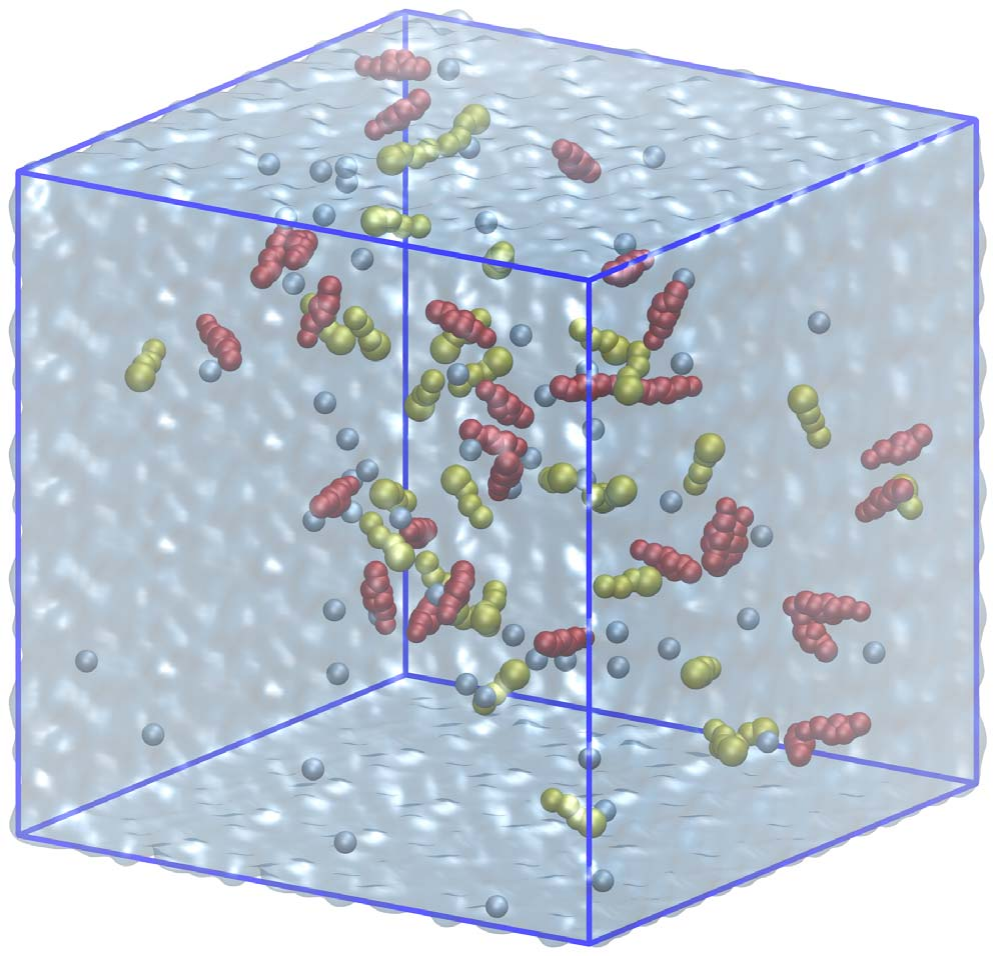
The initial structure of the coarse-grained molecular dynamics simulation: CA, SDS and Na+ are represented in red, yellow and blue van der Waals representation, respectively.

The aggregation of SDS and CA was studied by means of the Martini coarse-grained [17] molecular dynamics method (MD). SDS [16] and CA [18] molecules were modeled by 4 and 8 beads (interaction sites), respectively. 30 SDS and 30 CA were placed randomly into the simulation cell using the Packmol program package [19], [20]. In order to mimic the experimental conditions, appropriate number of water molecules (62000) was placed and 60 sodium cations were also included in the cubic box (**Figure 1**).

The calculations were performed with the Gromacs 4.5.4 program package [21]. After 2500 steepest descent minimization steps, the system was subjected to NPT pre-equilibration. In this part of the simulation, the step size was set to 4 fs and the calculation was 20 ps long. After the pre-equilibration, the time step was set to 20 fs and a 10 µs long NPT simulation was performed. During the MD calculation, the Berendsen temperature and isotropic pressure coupling scheme [22] was applied. The temperature was set to 27°C, the pressure to 1 bar, τ_T_ to 1.0 ps and τ_p_ to 3.0 ps in the pre-equilibration and 1.0 ps in the remaining part of the simulation. Periodic boundary conditions were applied in all 3 dimensions and a cutoff value of 12 Å was ascribed for the long-range interactions. A shifting function [23] was used for the van der Waals and electrostatic interactions from 9 Å and 0 Å, respectively. The volume of the simulation cell rapidly converged to 8114.11 nm^3^, and resulted in an effective surfactant concentration of 12.28 mM, which is far above the experimental *cmc* value.

## Results and Discussions

### Experimental investigations

**Figure 2:**
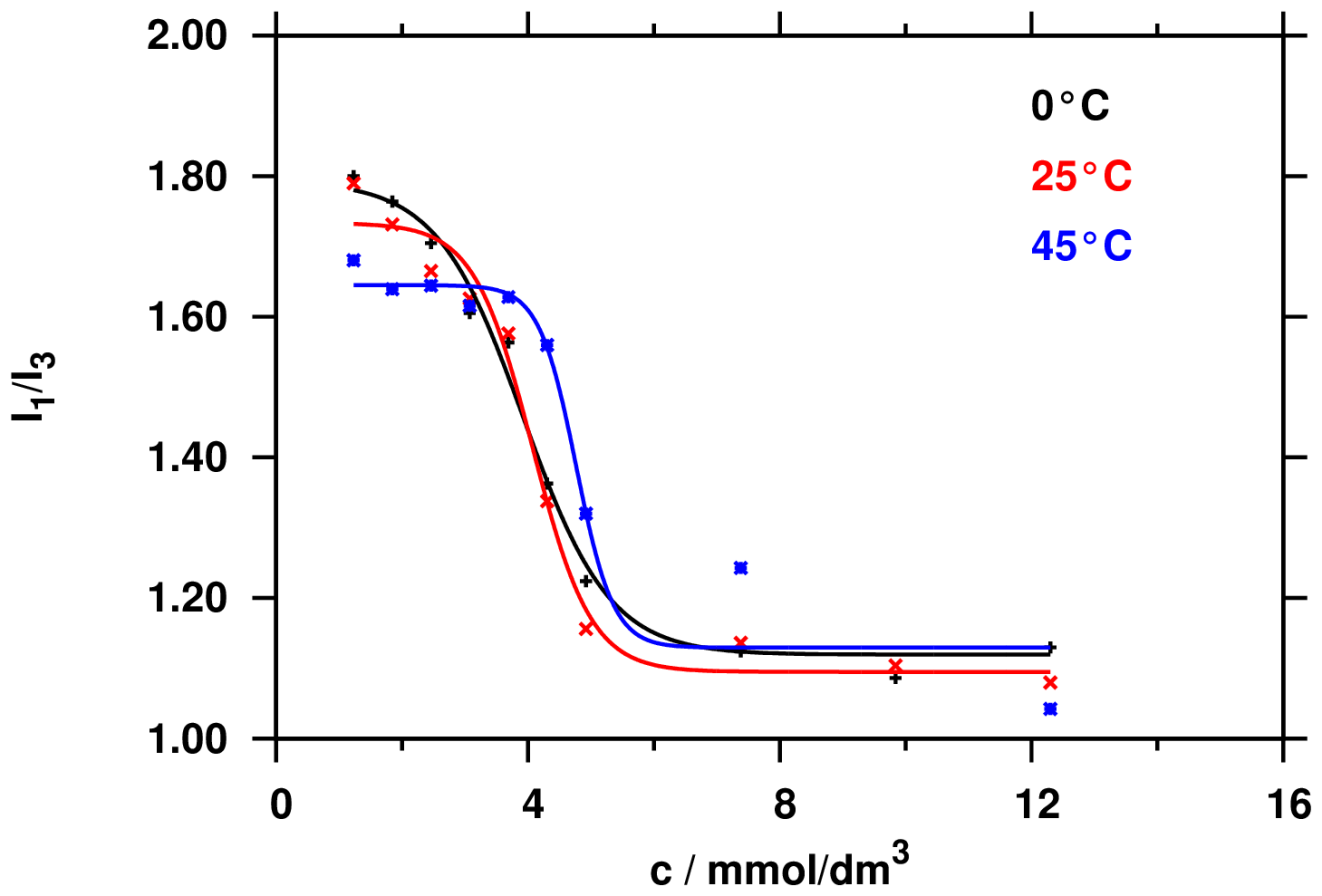
Change in the intensity ratio I1/I3 of pyrene as function of the total concentration of surfactants at different temperatures, cmc is determined by Boltzmann fitting.

According to the procedure described by Kim *et al*., a sharp change of the intensity ratio of the first and third vibronic emission bands of pyrene in the fluorescence measurements indicates the onset of the micellisation, causing the decline in the polarity of the pyrene microenvironment [24]. Therefore, this change in the I_1_/I_3_ ratio as a function of surfactant concentration (*c*) can be used to determine the critical micelle concentration. **Figure 2** shows representative results of the fluorimetric measurements for 1∶1 binary mixture of the NaCA and SDS at three different temperatures (0, 25 and 45°C). The temperature dependence of the fluorimetric *cmc* (*cmc^ex^_f_*) is weak in the range of 0 and 25°C, while the titration curve shifted to higher concentration values at 45°C resulting higher *cmc^ex^_f_* values.

All *cmc^ex^_f_* values measured in the temperature range of 0–50°C are tabulated in **Table 1** and agree to the critical micelle concentration obtained by surface tension measurement (***cmc^ex^_t_***) within the statistical error of 3.5% over the whole temperature range. It is worthy to mention that the *cmc^ex^_f_* values are tendentiously smaller than those of the tensiometric measurement.

**Table 1:** Temperature dependent critical micelle concentration obtained by fluorimetric and tensiometric experiments (noted by cmc^ex^_f_(T) and cmc^ex^_t_(T), respectively), ideal critical micelle concentration (cmc^id^), mole fraction of NaCA (*x_1_*) in the mixed micelle and interaction parameter between the building units (β_1,2_) as a function of temperature for the binary mixture of NaCA and SDS (1∶1).

t/°C	cmc_1,f_(T)/mM	cmc_2,f_(T)/mM	cmc^ex^_f_(T)/mM	cmc^ex^_t_(T)/mM	cmc^id^(T)/mM	x_1_	β_1,2_
0	9.00±0.16	10.00±0.12	4.09±0.12	4.19±0.05	9.47	0.508±0.015	−3.32
5	9.50±0.13	10.23±0.14	4.01±0.12	4.09±0.06	9.85	0.505±0.015	−3.56
10	10.00±0.18	10.47±0.17	4.01±0.12	4.12±0.06	10.23	0.503±0.015	−3.72
15	10.50±0.17	10.82±0.13	4.17±0.13	4.22±0.07	10.66	0.502±0.016	−3.74
20	11.00±0.21	11.31±0.18	4.12±0.12	4.23±0.08	11.15	0.502±0.015	−3.97
25	11.50±0.23	11.98±0.21	4.07±0.12	4.20±0.06	11.74	0.503±0.015	−4.23
30	12.00±0.25	12.40±0.22	4.21±0.13	4.33±0.07	12.20	0.502±0.015	−4.24
35	12.50±0.26	13.11±0.26	4.40±0.13	4.54±0.08	12.80	0.503±0.015	−4.25
40	13.00±0.29	13.58±0.31	4.54±0.14	4.70±0.09	13.28	0.503±0.015	−4.28
45	13.50±0.28	14.03±0.31	4.76±0.14	4.90±0.11	13.76	0.502±0.015	−4.22
50	14.00±0.35	14.78±0.35	6.78±0.20	7.02±0.16	14.38	0.504±0.015	−2.98

**cmc_1,f_ (T)**- cmc of pure NaCA measured by fluorimetry.

**cmc_2,_**_***f***_ ** ***(T)***- cmc of pure SDS measured by fluorimetry.

As we mentioned previously, the temperature dependence of *cmc* is weak over the temperature range of 0°C and 25°C. In this temperature range the formation of the micelle can be entropy driven, *i.e*. dehydration of apolar parts of building blocks [25], [26]. Between 30°C and 50°C the *cmc^ex^(T)* rises, which can be explained by the fact that motion of the monomers in the mixed micelle is more intensive with increasing the temperature, and it can destabilize the mixed micelle compared to those at lower temperatures.

**Figure 3:**
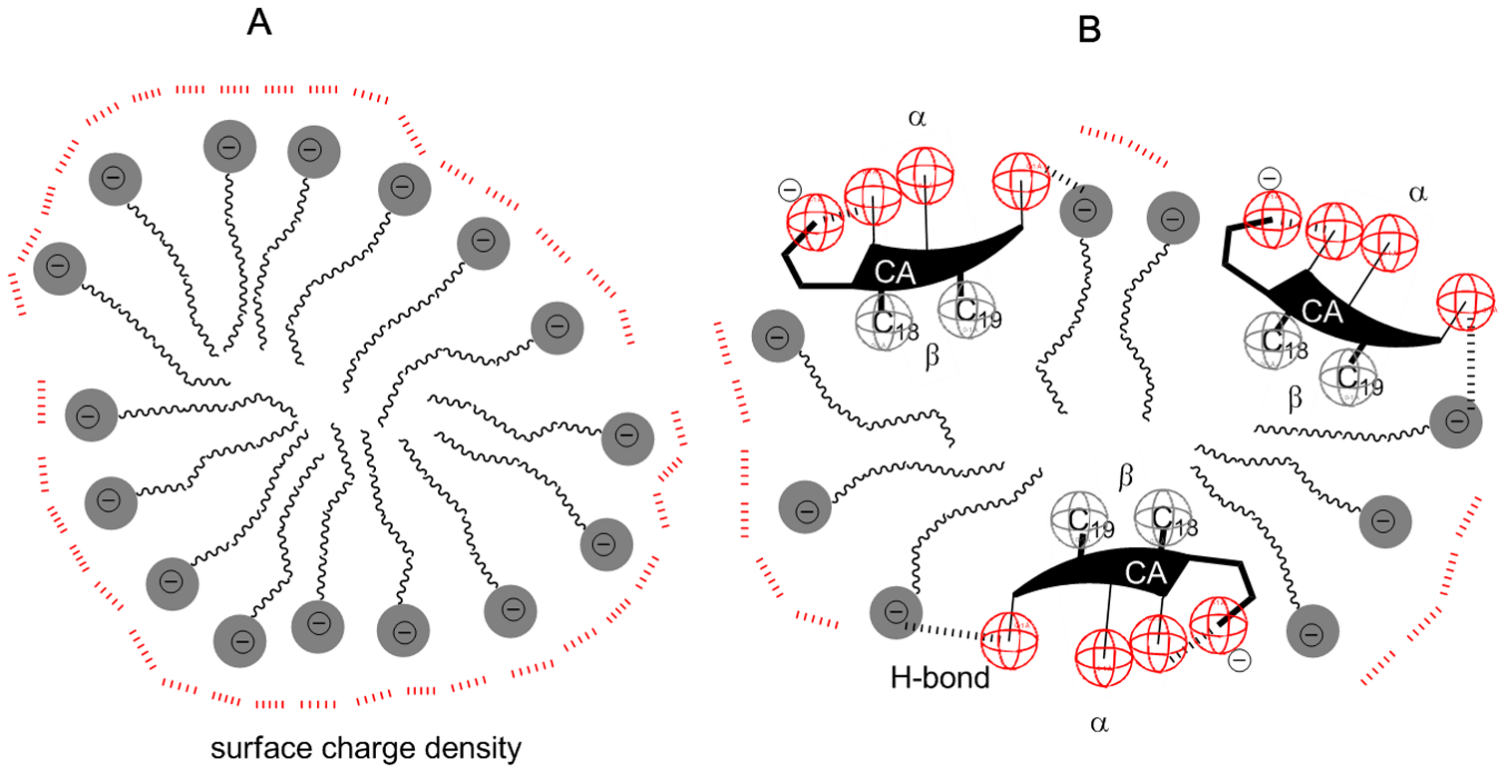
Micelle of pure SDS (A) and the mixed micelle of SDS and CA: the steroid skeleton of the CA anion breaks up the continuous surface density of electrical charge (B).

According to Clint [27], the temperature dependent ideal *cmc* (*cmc^id^(T)*) can be calculated for the investigated binary mixture from the *cmc* of the pure building units (*cmc_i_(T)*) obtained by fluorimetry measurement (*cmc_i,f_(T)*) at the appropriate temperature (T):
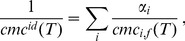
(1)where *α_i_* is the mole fraction of component *i* in the solution. As shown in **Table 1**, both *cmc^ex^_f_(T)* and *cmc^ex^_t_(T)* values are lower by at least a factor of 2 than the calculated *cmc^id^(T)* values at each investigated temperature. This suggests that the real mixed micelle of NaCA and SDS has a more exothermic enthalpy of formation compared to the ideal mixed micelle formation (for an ideal mixed micelle, micelle forming is exclusively entropy driven, without any change in enthalpy). Therefore, formation of the real micelle is more favorable than that of the ideal mixed micelle due to negative excess free energy (Δ_excess_G<0). According to Rubingh's equation [28], the mole fraction (*x*_1_) of the more hydrophobic building unit (NaCA) in the mixed micelle can be estimated using the following equation:
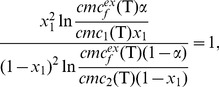
(2)Where *cmc*_1_ *(T)* is the critical micelle concentration of the pure NaCA (the most hydrophobic component), while *cmc*_2_ *(T)* is the critical micelle concentration of pure SDS. It is interesting to mention that *x_1_* is rather insensitive to the temperature change in the present study (**Table 1**). By knowing the parameters discussed above, the interaction parameter between the building units (*β_1,2_(T)*) can be calculated [28] as follows:
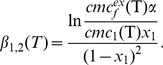
(3)The interaction parameter is negative in the whole temperature range studied, suggesting the existence of synergistic effect (additional attractive interactions between different building units). Since both building blocks of the mixed micelle are anionic, it would be expected that there are repulsive electrostatic interactions between them (positive values of the interaction parameter represent the antagonism between the building units (Δ*_excess_G>*0)). The negative value of the interaction parameter can be explained by the structure of the mixed micelle. As it can also be seen from the detailed analysis of the MD simulation, the shape of these mixed micelles are almost spherical, and the anions of the bile acids can fit between the SDS polar heads at the surface of the micelle (see **Figure 3**). The concave side of the steroidal skeleton (with the OH groups) can face towards the bulk solution, while the convex side with the angular methyl groups is oriented towards the hydrophobic domain of the aliphatic strains of SDS. The presence of the steroid skeleton with the relatively large surface on the interface of the mixed micelle fills the gap between the sulfate groups of SDS. Two CA anions can also form hydrogen bonds between the carboxylate group and the C_12_ OH group [1], and therefore the carboxylate group is masked from the repulsive interactions. High negative values of the interaction parameter show possible hydrogen bonds between different building units as well. These bonds are possible between the axial (compared to the steroid skeleton) OH groups of cholic acid and sulfate groups of SDS (**Figure 3**).

The lowered interaction parameter at 50°C (**Table 1**) might indicate the increased intensity of the motion of the building blocks in the micelle [29] and this leads to the partial break of the hydrogen bonds.

Besides the interactions between the micelle building units, the binding of the counterion to the micelle also plays important roles in the micelle formation. Such information can be accessed via the surface charge density which can be measured by specific conductivity (*κ*) as function of the total concentration of the surfactants (*c*).

**Figure 4:**
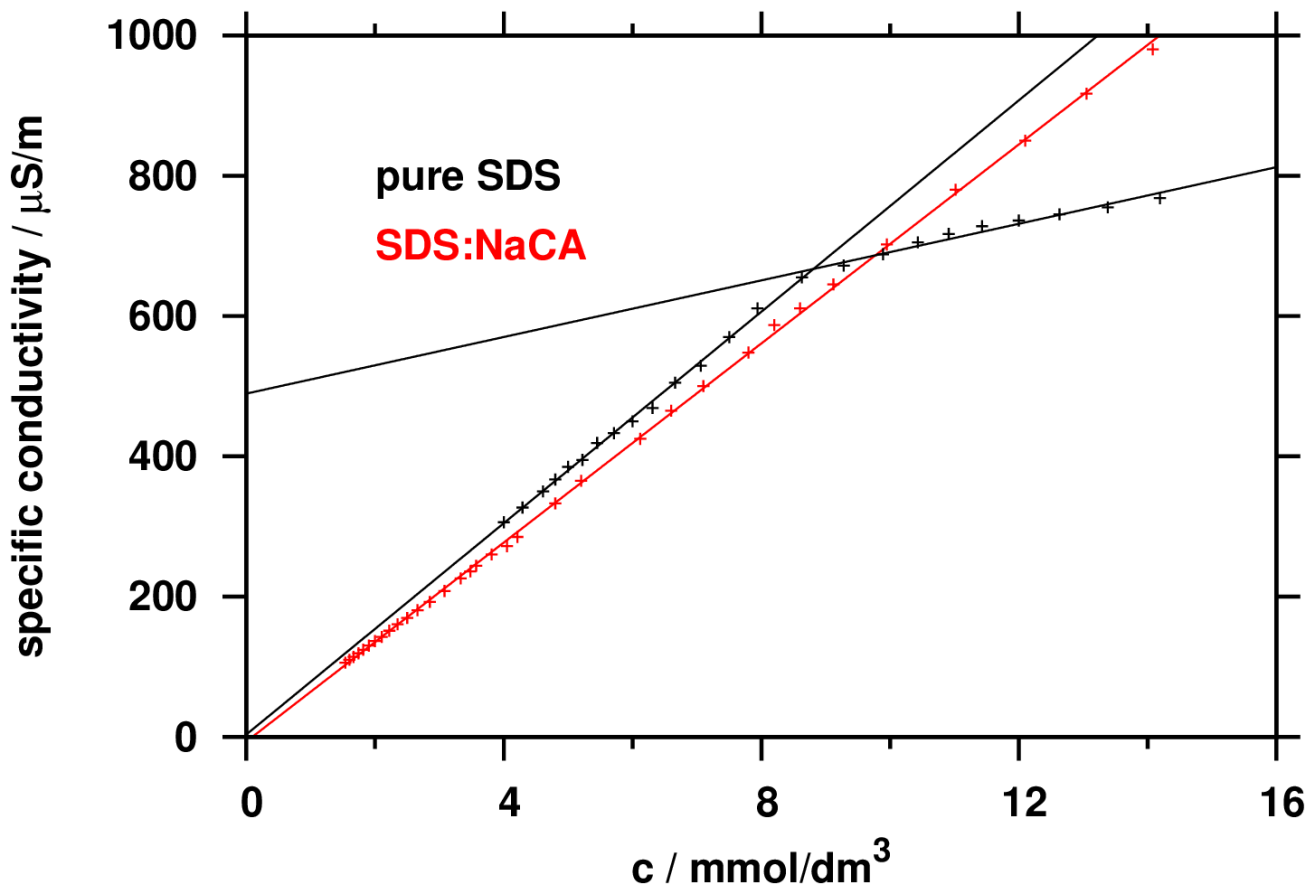
Change of specific conductivity as function of surfactant concentration.

As **Figure 4** shows, a distinct breakpoint found for pure SDS, indicating the formation of the micelle at concentration of 8.8 mM. Transition in the specific conductivity is due to the fact that the sodium counterions at the micelle surface partially neutralize the net electrical charge of the micelle, and therefore its mobility decreases compared to the state before micellisation, when all monomers were ionized. As it seen in **Figure 4**, there is no such break point of *κ*(*c*) for the 1∶1 binary mixture of NaCA and SDS. This means that after the formation of the micelle the total negative charge is free, the negative charges of the micelle are not neutralized by the oppositely charged ion. This confirms the assumption that the steroid skeleton, because of its size and capacity of hydrogen bonding and charge screening, prevents the forming of the compact negative surface charge.

### Theoretical study

**Figure 5:**
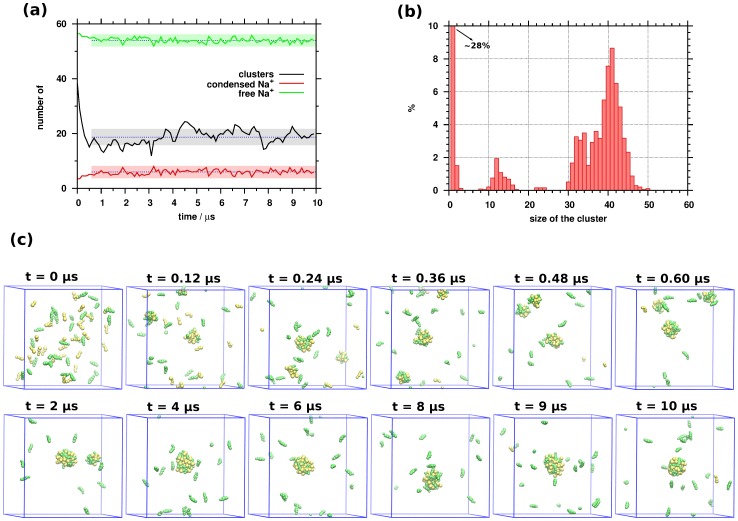
(a) Time evolution of the number of clusters (NOC), free and condensed Na+ cations in the course of the molecular dynamics simulation applying block averages using 100 ns intervals. Dashed lines represent the mean and the transparent bands the standard deviation (values were calculated from 0.6 µs to 10 µs). (b) The distribution of the cluster size. (c) Selected frames from the molecular dynamics trajectory at different time intervals.

Macroscopic micelle properties can only provide limited knowledge of the molecular interactions using classical thermodynamic models and derived descriptors are average over different micelles. Coarse-grained simulation can help to rationalize these experimental results and they are able to extend the understanding at near molecular scale. For instance, association of surfactant molecules can be determined using the number of the clusters (NOC) formed (**Figure 5/a**).

We consider two or more molecules as a cluster if the distance between their closest beads was less than 7 Å. At the beginning of the simulation the NOC was 60, since all the surfactants were set up randomly. A drop of NOC was obtained until 0.60 µs indicating the association of the monomers. After this period, equilibrium was obtained, where the NOC value fluctuates between 9 and 27 with an average NOC of 18.7 (±2.8). The average NOC shows that 18.7 different clusters can be found in each frame in average, but their cluster size and decomposition can be varied. As **Figure 5/b** shows the distribution of the cluster size is basically trimodal and the maximum of these peaks are located at 1, 12 and 41 molecules. The second peak of the cluster size distribution curve is due to the fact, that before 3 µs smaller aggregates (with cluster size of 12±3, 23±1 and 33±3 monomers) were formed mostly. After 3 µs we obtained larger micelles (by self-assembly of the smaller aggregates, with cluster size of 41±4) and individual surfactants. The cluster size distribution curve was calculated between 0.6 µs and 10 µs because the NOC value stabilized in this range therefore the population of smaller micelles is visible on the distribution curve (time evolution of cluster size distribution is available as animated gif in Figure S1). During the further analysis we used the same time range because we were interested in the whole process of the micelle formation. Roughly a third of the monomers (28.7%) do not aggregate during the simulation. These individual molecules are almost exclusively CA molecules (∼98%), as the last snapshot of **Figure 5/c** shows. The process of the aggregation can also be seen in **Figure 5/c**. Until ∼2 µs only small aggregates were formed and these clusters were further assembled into one larger micelle after 4 µs. It is important to mention that the largest micelle has a dynamic structure, since mobile CA molecules leave or join to the micelle. Based on the histogram in **Figure 5/b**, the cluster with 41 surfactant molecules is the most dominant (>8%), which mainly consists of SDS. The SDS content of the micelles is 66.6±7.8% averaged over all aggregates along the 9.4 µs long equilibrated trajectory (*x*_2_ = 0.66±0.08).

**Figure 6:**
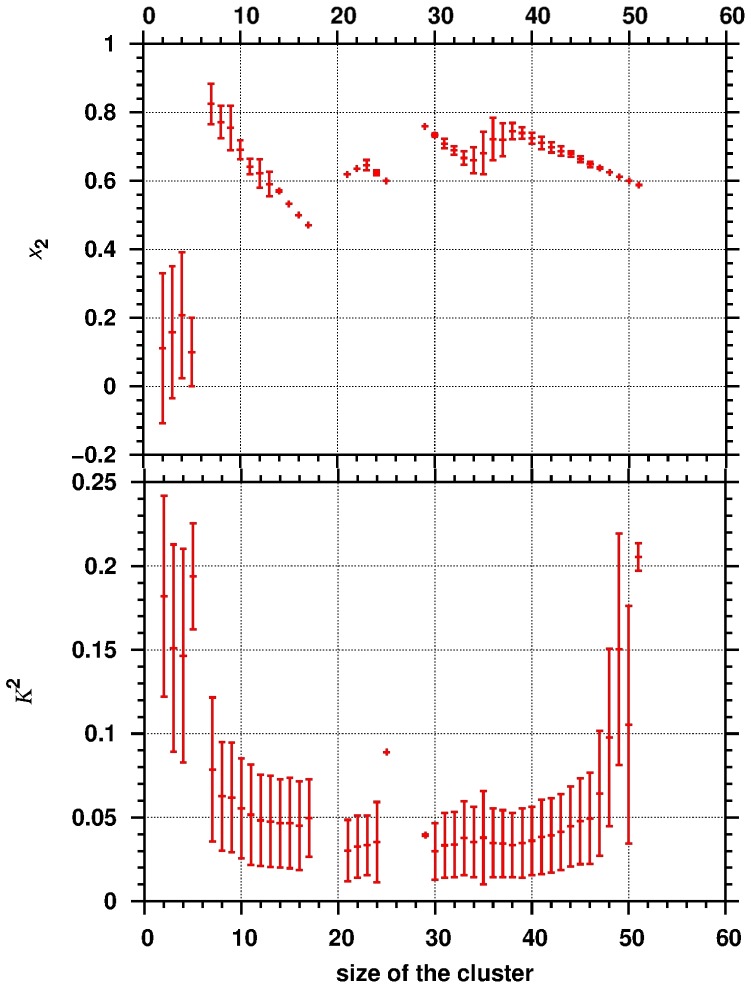
The average SDS content (x2) of the micelle (upper panel) and their relative shape anisotropy (lower panel, Κ2) as a function of the cluster size.

As the upper panel of **Figure 6** shows, the cluster size dependence of the SDS content was also determined. For small instable aggregates (consisting of 2, 3, 4 or 5 surfactants) the average SDS content is low and scattered, while high SDS content is found in the range of 7–17 cluster size, near the second peak in the cluster size distribution in **Figure 5/b**. The 80 percent average SDS content (as well as standard deviation) at cluster size of 7 decreases linearly with increasing cluster size and standard deviation becomes rather small for aggregates with 14 surfactants. The larger deviation in the SDS content for smaller aggregates (7–10) can indicate that the joining of individual CA molecules are statistically preferred more than that of SDS, on the other hand either CA or SDS compounds are able to enter or leave the aggregates. Reduction of the standard deviation can suggest that a stable SDS core was formed and the cluster size is increased due to joining CA molecules. Similar behavior of the micelle SDS content was obtained in the cases of the clusters around aggregation number of 41 (**Figure 6**). The highest abundance of SDS (x_2_≈0.75) in the micelle is found at aggregation number 38. Micelles with higher cluster size contain less SDS compared to the cluster at 38 of aggregation number due to incoming CA molecules. Consequently, the average SDS content decreased linearly with the cluster size. These data shed some lights on the process of micelle formation: first a stable SDS core forms with a certain number of surfactants, and then CA molecules adsorb on the surface of the micelle.

The calculated SDS content of the micelles (x_2_≈0.7) is higher by 0.2 than the experimental value shown in **Table 1**. We suppose that the limited duration of the MD simulation might be not enough to obtain thermodynamic equilibrium. However, it is important to keep in mind the following: As stated earlier, a core structure is forming and thereafter the unbound CA molecules enter this core structure and thus increase the size of the cluster. As shown in the snapshots of **Figure 5/c**, after 4 µs a larger micelle is composed by almost all available SDS molecules and the mobile CA can enter and leave this cluster dynamically (this is indicated by the varying size of the cluster over time). The structure of the core micelle is not able to receive more CA compounds; therefore a structure alteration should occur on the core structure in order to bound more and more CA molecules. Our simulations capture the first steps of the micelle formation and can give a semi-quantitative picture about the process of aggregation of SDS and CA.

The accuracy of force field parameters could also influence on the composition and structure of micelles. The parameters used in this study had been successfully used in other studies [16], [18] and are in good agreement with experimental results.

In order to characterize the shape of the micelles formed, the relative shape anisotropy (*Κ* ^2^) was calculated (lower panel on **Figure 6**) according to the following equation:
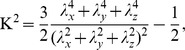
where λ_x_, λ_y_, λ_z_ are principal moments. If *Κ* ^2^ = 0 then the shape is spherical symmetric and if *Κ* ^2^ = 1 the structure is linear. As **Figure 6** shows, highly anisotropic aggregates are preferably formed for small (<6) and large (>49) cluster sizes, while almost spherical micelles (K^2^≈0.05) are observed for middle-sized clusters. For smaller aggregates it is obvious that they cannot form spherical structures because of the small number of surfactants. For larger aggregates the observed high anisotropy is due to the fusion of two smaller micelles.

Standard counterion condensation theory suggested by Manning [30], [31] can be used to explain the counterion condensation on systems with different micelle shape such as charged spheres, cylinders, and planes [32], [33]. According to this theory, if the counterion concentration is small but higher than zero, there are ions close to the charged spheres (or condensed on the spheres) and there are ions in the bulk phase [32]. As it was demonstrated in the conductivity measurement, the micelle-bound Na^+^ counterion can neutralize the surface charges of the micelle originating from the negatively charged SDS and CHOA (charges represented by SO3 and OCO beads in the simulation). Such Na^+^ ions (so-called condensed cations) can also be found within the distance of 7 Å from the negatively charged SO3 and OCO beads in the simulation. As one can see in **Figure 5/a**, 10% of Na^+^ are such condensed cations (the average number of condensed and free cations is 5.9±2.1 and 54.0±2.1, respectively). However, direct comparison between our results and that of obtained by Manning *et al*. is not straightforward since the micelles formed are close to spherical, but for the anisotropic shapes qualitatively similar results were obtained.

**Figure 7:**
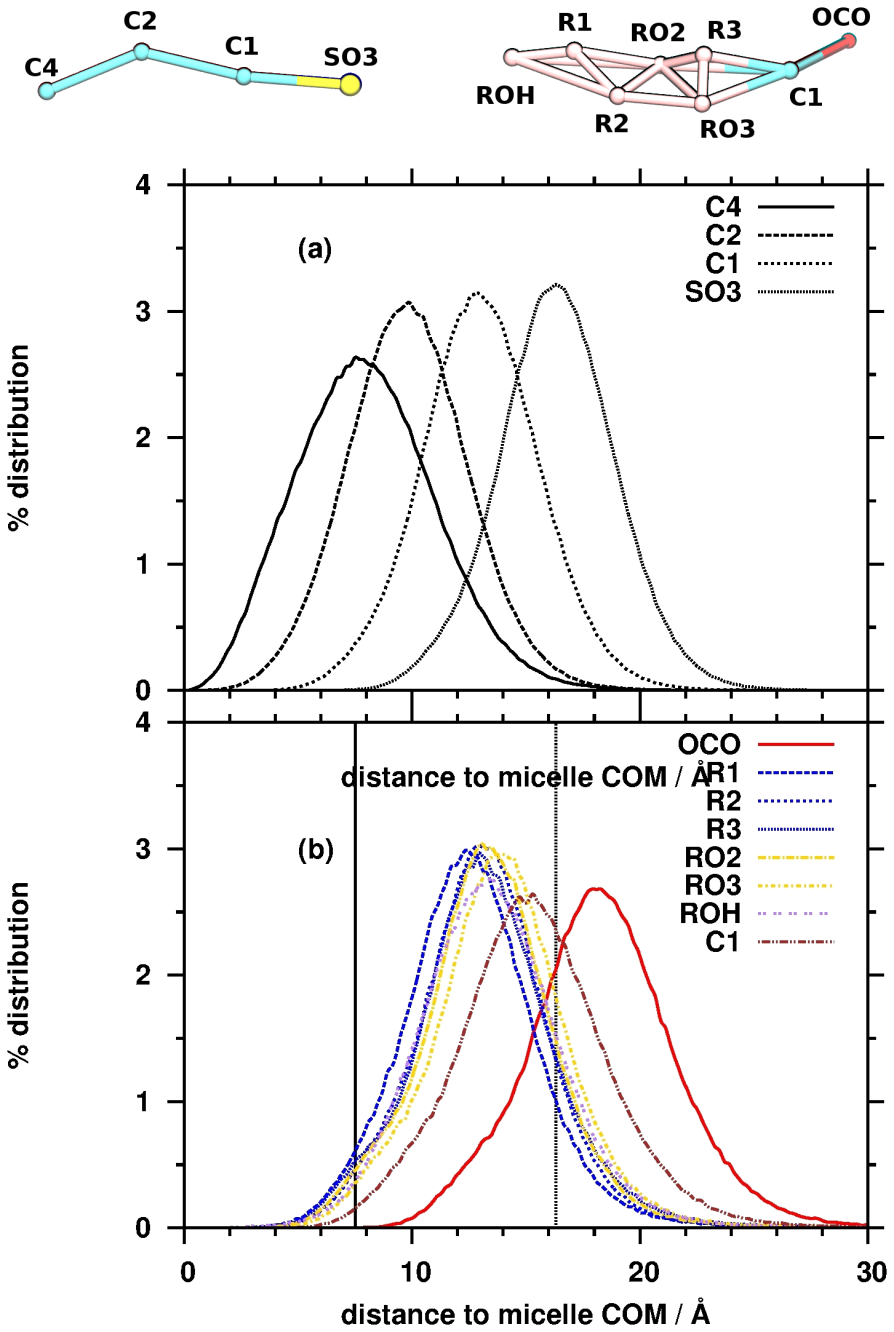
Radial distributions of the various interaction sites of SDS (upper panel) and CA (lower panel) surfactants. On the lower panel the first and the second vertical lines indicate the position of peak maxima of C4 and SO3 beads of SDS, respectively.

To get a consistent picture about the structure of mixed micelles, we focused our analysis on the radial distribution of the various interaction sites inside the micelles formed (**Figure 7**).

**Figure 8:**
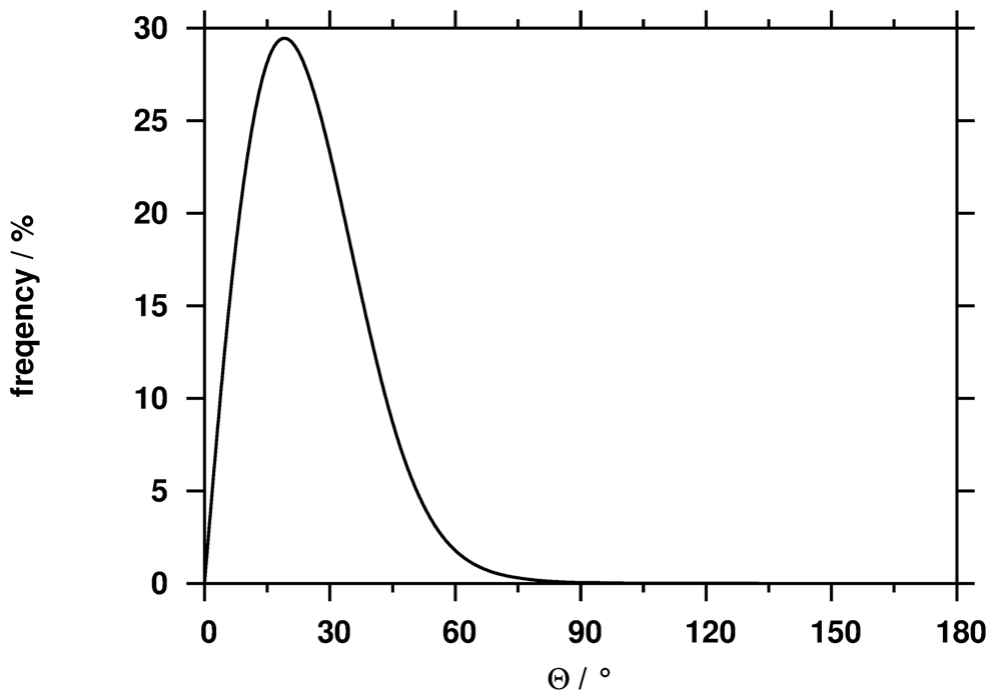
Distribution of the angle between 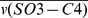
 **and** 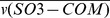 **vectors.**

Considering that the C4 beads have a peak between 6–8 Å, C2 beads between 8–10 Å, C1 beads between 12–14 Å and SO3 beads around 16 Å, we can conclude that the SDS surfactant molecules are oriented with their hydrophobic part towards the COM. The highly hydrophilic part (SO3 beads) pointed toward the water phase, where favorable interactions can form between the SDS and water molecules. The distribution of C4 bead of SDS is significantly broader compared to that of C2, C1 and SO3. There are two reasons for this behavior: larger flexibility inside the micelle core as suggested by Jalali *et al*. [16] and not all SDS molecules pointed toward the micelle COM. The orientation of the SDS molecules inside the micelle was characterized by calculating the angle (Θ, **Figure 8**) between two vectors: 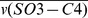 and 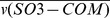. If the Θ angle close to zero then the SDS orientated toward the COM along the radial of the micelle. As one can see in this figure, we obtained not only Θ values close to 0, but there are a number of SDS molecules where the Θ value is larger than 45°. This deviation resulted in the fact that C4 beads are getting closer to the surface and having overlap with C1, C2 and SO3 beads in the micelle.

**Figure 7/b** shows the distribution of CA beads. The distributions of the hydrophobic and slightly hydrophilic parts of the CA molecules coincide and they are situated in a kind of intermediate layer of the micelles (located mainly between the C2 and SO3 beads). In contrast to this, the distribution of the C1 bead is slightly shifted toward the highly hydrophilic OCO being on the surface of the micelles. This suggests that the CA molecules are on the hydrophobic surface instead of pointing towards the COM of micelles and this arrangement gives rise to the hydrophilic interaction with the polar solvent. Furthermore, due to the fact that hydrophobic beads (R1, R2, and R3) are located deeper compared to hydrophilic beads (RO2, RO3, and ROH) resulted in increased interaction: hydrophobic beads interacted with the hydrophobic core of SDS and hydrophilic beads orientated toward the water phase. These structural features are in good agreement with the conclusion and interpretation of the conductometric measurement (see **Figure 3**).

## Summary

Mixed micelles in the binary mixture of NaCA and SDS (1∶1) are thermodynamically more stable than their ideal mixed micelles due to a synergistic effect (*β*_1,2_<0) in the mixed micelles over the whole temperature range studied (from 0°C to 50°C). The mixed micelles are practically totally ionised, the binding of the oppositely charged ions is negligible. The results of the coarse-grained molecular dynamics simulations are in semi-quantitative agreement with the experimental results. Using the theoretical calculations we confirmed the shape of the micelle, and the distribution of the various components within the micelles formed. In addition we proposed a possible micelle formation mechanism.

## Supporting Information

Figure S1 **Time evolution of cluster size distribution.**
(GIF)
