# Supplementary figures and images for: Mixed Micelles of Sodium Cholate and Sodium Dodecylsulphate 1:1 Binary Mixture at Different Temperatures – Experimental and Theoretical Investigations
Source: PLoS One. 2014 Jul 8;9(7):e102114. doi: 10.1371/journal.pone.0102114 (PMC4087020; doi:10.1371/journal.pone.0102114)

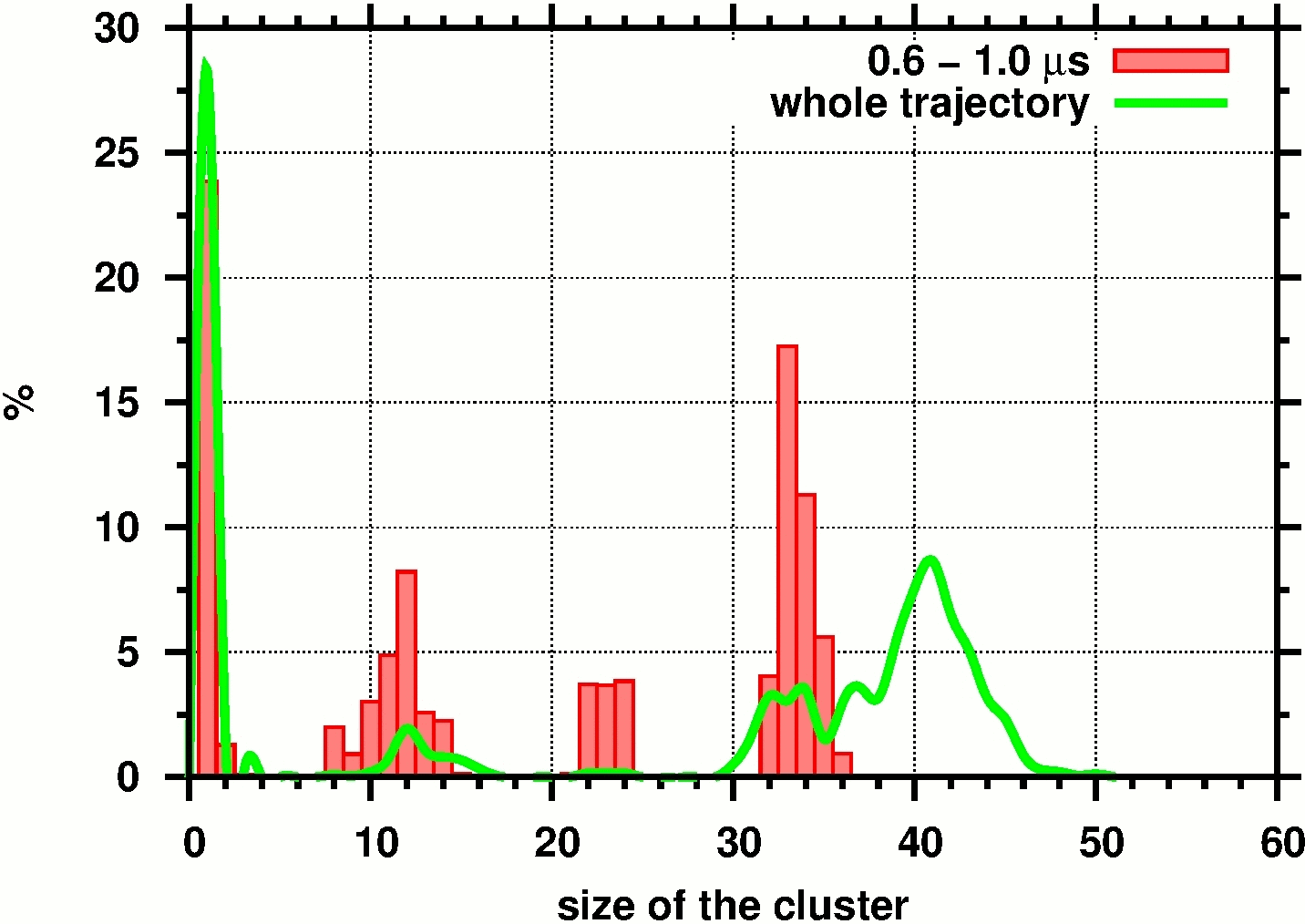

Supplement: Figure S1 — Time evolution of cluster size distribution. (GIF) [file pone.0102114.s001.gif]
